# Effect of deep margin elevation with different injectable materials on performance of CAD/CAM-Fabricated nanoceramic-resin onlays: 3-year randomized clinical trial

**DOI:** 10.1007/s00784-025-06705-7

**Published:** 2026-01-07

**Authors:** Basema Nader Roshdy, Radwa Ibrahim Eltoukhy, Ashraf Ibrahim Ali, Salah Hasab Mahmoud

**Affiliations:** 1 Faculty of Dentistry, Conservative Dentistry Department, Horus University, New Damietta, Egypt; 2 Faculty of Dentistry, Conservative Dentistry Department, Mansoura University, Mansoura, Egypt

## Abstract

**Objective:**

To evaluate the effect of deep margin elevation (DME) with four different injectable restorative materials on the three-year clinical performance of nanoceramic-resin CAD/CAM onlay restorations.

**Materials and methods:**

Sixty-four adult patients with subgingival, broad Class II carious molars indicated for onlay restorations were randomly assigned to four groups according to the injectable DME materials used: highly viscous glass ionomer (HVGI), high-filled injectable composite resin (ICR), resin-modified glass ionomer (RMGI), and bioactive ionic resin (BAIR). The proximal subgingival margins were repositioned to a supragingival level using the assigned materials; subsequently, all groups received nanoceramic-resin CAD/CAM onlay restorations. Gingival health and restorative performance were assessed over a three-year evaluation period using the Gingival Index (GI) and modified World Dental Federation (FDI) criteria. The collected data were statistically analyzed.

**Results:**

All restorations were evaluated with a 100% recall rate and a 100% survival rate. For GI, assessment of scores across various evaluation periods within each group revealed a significant increase over time. Nevertheless, no significant differences were found among the tested groups at each evaluation period. Additionally, FDI evaluation criteria showed no significant differences.

**Conclusions:**

After three years, nanoceramic-resin CAD/CAM onlay restorations following deep margin elevation with injectable restorative materials demonstrated acceptable clinical behavior, with only a slight increase in gingival bleeding.

**Clinical relevance:**

Injectable restorative materials can provide adequate clinical performance as deep margin elevation materials for molars restored with nanoceramic-resin CAD/CAM onlays.

**Supplementary Information:**

The online version contains supplementary material available at 10.1007/s00784-025-06705-7.

## Introduction

The difficulty in managing subgingival proximal carious lesions in posterior teeth constitutes a significant hindrance to achieving durable adhesive restorations. This challenge arises mainly from difficulties in moisture control and restorative technicalities, particularly when indirect restorations are indicated for extensive lesions that weaken the tooth structure. A commonly adopted approach to overcome this problem is the surgical exposure of deep cervical margins through apical displacement of periodontal tissues, known as surgical crown lengthening. Although this approach preserves periodontal health and respects the biological width, it may result in attachment loss and anatomical complications, such as the exposure of root concavities or furcations [1, 2].

As a less invasive alternative, the promising approach of Deep Margin Elevation has been introduced to address these challenging clinical scenarios. This technique involves repositioning deep cervical margins to a more favorable supragingival location using a direct restorative material, thereby simplifying subsequent restorative procedures. By achieving better isolation, DME facilitates critical clinical steps essential for long-term success, including impression recording and cementation. Conversely, inadequate isolation may compromise the marginal seal, leading to secondary caries and periodontal complications [3–5].

The selection of the most suitable material for managing subgingival and extensive defects has received considerable attention [6–9]. Injectable restorative materials have recently emerged as alternatives to conventional DME materials [5, 7, 10], aiming to streamline the clinical workflow and improve adaptation to irregular surfaces. Initially, the DME concept involved using conventional glass ionomer cement at the base of the proximal cavity. More recently, favorable clinical outcomes have been reported with resin-modified glass ionomers and resin composites as DME materials [11]. Contemporary high-filled, low-viscosity resin composites have been employed, offering mechanically superior alternatives to conventional flowable composites [12, 13]. With the growing emphasis on bioactivity, bioactive ionic resins have been investigated for their potential use as DME materials [7, 9]. Additionally, nanoceramic-resin CAD/CAM restorations provide an overlying indirect esthetic restoration with adequate resiliency, biomimetic resistance to wear [14–16], satisfactory clinical performance, and easier adjustments if needed [17, 18].

Dental practitioners require robust scientific evidence from clinical trials to determine whether the use of different injectable restorative materials in DME preceding indirect restorations is justifiable. Although laboratory investigations play a crucial role in the early assessment of dental restoratives [19], clinical studies are necessary to account for the numerous patient-specific variables that influence overall performance. These variables include masticatory forces, abrasive meals, chemically active foods and fluids, temperature fluctuations, humidity variation, bacterial byproducts, and salivary enzymes [20]. However, only a few clinical trials have investigated DME with different injectable materials prior to indirect restorations [5, 21, 22]. Identifying the optimal DME material, particularly in terms of its effects on periodontal tissues and clinical performance, remains a significant clinical concern [4, 23–28]. Therefore, this clinical trial was designed to evaluate the effect of DME using different injectable restorative materials (HVGI, ICR, RMGI, and BAIR) on the three-year clinical performance of nanoceramic-resin CAD/CAM onlay restorations. The null hypothesis assumed that the clinical performance of nanoceramic-resin CAD/CAM onlay restorations wouldn’t be affected by DME using different injectable restorative materials.

## Materials and methods

### Materials

Four different injectable restorative materials were used to elevate deep sub-gingival margins: a highly viscous glass ionomer (Equia Fil, GC, Tokyo, Japan), a high-filled injectable composite resin (G-aenial Universal Injectable, GC, Tokyo, Japan), a resin-modified glass ionomer (GC Fuji II LC, GC, Tokyo, Japan), and a bioactive ionic resin (Activa™ Bioactive Restorative, Pulpdent, Watertown, MA, USA). CAD/CAM nanoceramic resin-based onlays, using Grandio Blocs (VOCO GmbH, Cuxhaven, Germany), were constructed and cemented to restore the teeth following DME.

### Ethical approval and study design

This study is a prospective, randomized, clinical trial employing a multi-arm, parallel design in accordance with the CONSORT guidelines [29]. The protocol was approved by the Dental Research Ethics Committee at Mansoura University (M10060721) and subsequently registered in the clinical trials database (www.clinicaltrials.gov) under the identification number NCT06155773. The restorative procedures were conducted from August 2021 to January 2022, whereas the follow-up was completed in February 2025.

### Sample size calculation

The sample size was calculated using Power Analysis and Sample Size (PASS) Software (version 15, 2017; NCSS, LLC, Kaysville, Utah, USA). Based on the Gingival Index scoring (0–3) and a large effect size (W = 0.5) [26], a sample size of 64 has achieved 81% power with 9 degrees of freedom using the chi-square test at a significance level (α) of 0.05.

### Patient selection and eligibility criteria

**Fig. 1:**
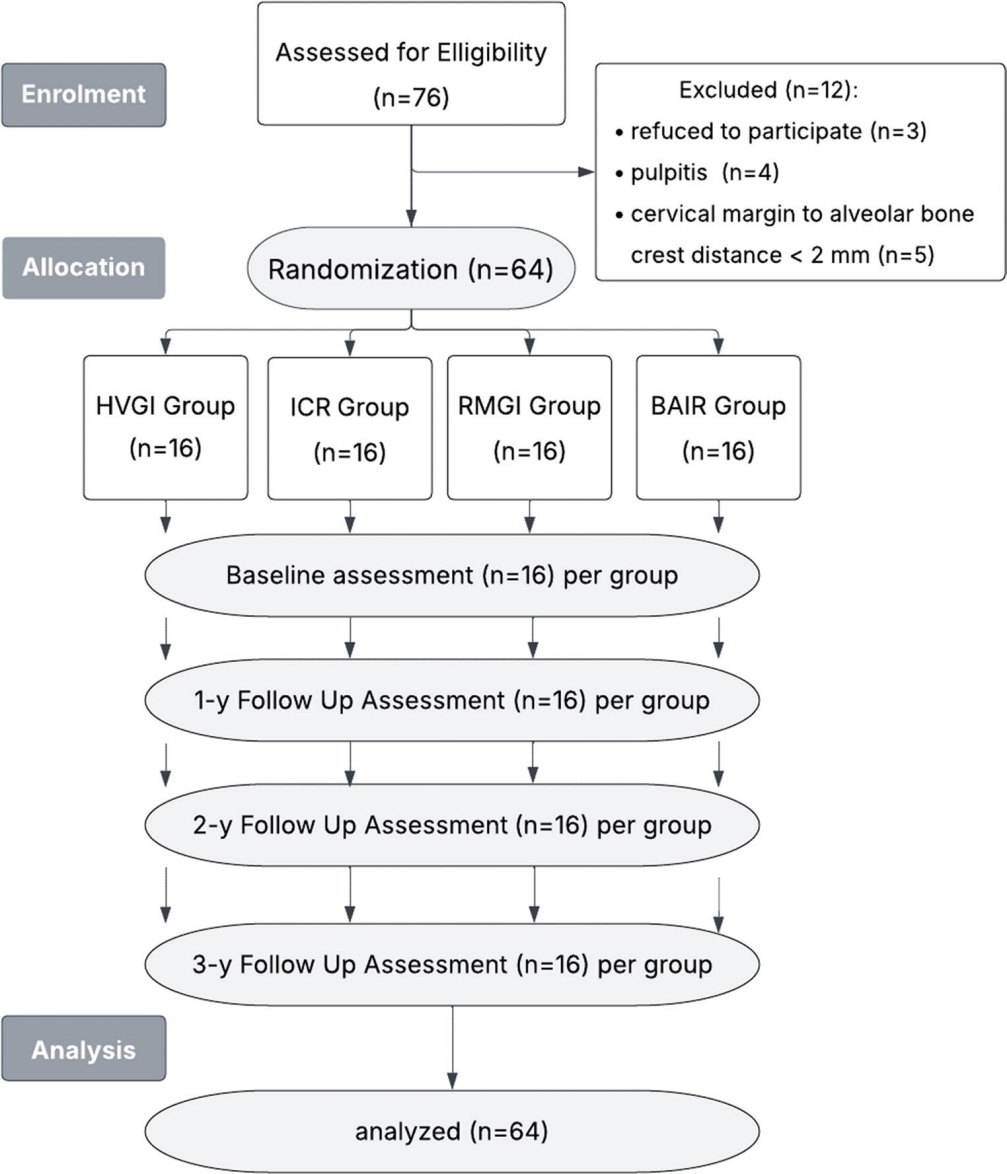
Clinical study design flowchart

Seventy-six patients were assessed to determine eligibility (Table 1) through the outpatient clinic at the Faculty of Dentistry, Mansoura University. Each underwent a comprehensive evaluation, including a medical and dental history, a focused clinical and radiographic examination, electric pulp vitality testing (DY310, Denjoy, Hunan, China), and a study cast analysis. Sixty-four participants met the inclusion criteria and provided informed consent after receiving detailed explanations of the study procedures. They received prophylactic periodontal and hygiene treatments and were encouraged to maintain optimal oral hygiene throughout the study. Participants were randomly assigned to four groups (*n* = 16), as illustrated in the clinical study design flowchart (Fig. 1).

**Table 1:** Eligibility criteria of the study

Inclusion criteria	Exclusion criteria
• Molar tooth with proximal subgingival carious lesion of ICDAS 4 or 5 causing weakening of one or more cusps. • Age: 20–40 years • Good General health • Normal alignment with the adjacent and opposing teeth. • Acceptance of follow up period and recall visits for forty-two months.	• Distance between the cervical margin and the crestal bone < 2 mm • The tooth would require direct pulp capping. • Poor oral hygiene (or not completing the oral hygiene phase). • Chronic periodontitis. • Pulpitis or non-vital tooth. • Teeth acting as abutment for fixed or removable prosthesis • Orthodontic treatment. • Parafunctional habits. • Systemic disease • Pregnancy

### Random sequence generation and allocation concealment

A simple randomization list was generated using an online tool (https://www.randomizer.org). The randomized treatment codes (HVGI, ICR, RMGI, or BAIR) were placed in sealed, opaque envelopes, which were not opened until tooth preparation. The randomization list and envelope preparation were conducted by a staff member who was not involved in any phase of the clinical trial.

### Blinding

This clinical trial was conducted as a double-blinded study. Neither the participants nor the clinical examiners were aware of the type of restorative intervention being used.

### Clinical procedures

A single experienced operator performed all restorative procedures under 3.5× magnification (Rose Micro Solutions, West Seneca, New York). A split-dam technique was applied using a rubber dam kit (KSK DENTECH, Tokyo, Japan) and a rubber non-latex sheet (silk blue, Dental Dam, Sanctuary). The concept of complete caries removal was adopted for preparing the external cavity walls utilizing a high-speed handpiece with copious water coolant. In contrast, selective caries removal concept was applied to the internal walls near the pulp using a sharp excavator (Maillefer, Dentsply, Switzerland).

A digital periapical radiograph was taken to evaluate the remaining dentin thickness in deep areas and the proximity of the cervical margin to the bone crest. The rubber dam sheet was then replaced. In cases of persistent bleeding, a piece of sterilized Teflon tape (Polytetrafluoroethylene, PTFE, Mishoo^®^, Linan Linfeng Fluorine, Plastics Co., Ltd., Hangzhou, Zhejiang, China) was placed between the gingival margin and the rubber dam sheet to ensure proper isolation. Cervical margin exposure was achieved using different methods, based on the depth of the margin. Cases of mild depth benefited from the application of the rubber dam and Teflon. Conversely, margin acquisition for deeper cervical margins was facilitated by a Therma-cut bur (no. 012, Therma-Cut, Dentsply Maillefer GmbH, Germany) with a high-speed contra-angled handpiece without water coolant [30].

#### Onlay preparation

All cusps were assessed using a caliper (Coltene/Whaledent AG), and those with a remaining thickness of less than 2 mm were capped. Weak cusps were reduced by 1.5 mm following the occlusal surface inclination with a butt-joint preparation [31, 32]. The onlay cavity was prepared and finished using an Inlay Set (Intensiv, Viganello-Lugano, Switzerland).

#### Deep margin elevation 

A circumferential band (Slick Bands™ Tofflemire-Style Kit, Garrison Dental Solutions, MI, USA) was used to elevate the cervical margins. The band’s adaptation to the cervical margin was carefully verified using magnification loupes, ensuring that no rubber dam or gingival tissue was trapped. Cervical margins with concave geometry or unsealed bands required the Matrix-in-Matrix technique [33]. The DME material was applied according to the study group guidelines and the manufacturer’s instructions. The injectable DME materials were carefully dispensed into the proximal cervical portion to avoid air entrapment, with injections made close to the margin. A dental explorer (Maillefer, Dentsply, Switzerland) was then used along the line angles to remove any trapped bubbles under magnification.

#### Immediate dentin sealing

For all groups, exposed dentin surfaces were sealed using a universal adhesive (G-Premio BOND, GC, Tokyo, Japan) and subsequently light-cured at an intensity of 1470 mW/cm^2^ using Elipar™ Deep Cure-L LED Curing device (3 M ESPE, St. Paul, MN, USA). A thin layer of flowable composite (G-aenial Flo X, GC, Tokyo, Japan) was then applied to support the sealed dentin surfaces [34, 35]. The surfaces of the cervical restorations and sealed dentin were light-cured under a layer of glycerin gel to eliminate the oxygen-inhibited layer. Enamel margins were subsequently refined, and the sealed dentin surfaces were inspected under magnification loupes [26, 36].

#### Restorations fabrication

After scanning the prepared cavity, the provisional restoration was directly fabricated with the aid of a waxed study cast using Structure 2 Sc (VOCO GmbH, Cuxhaven, Germany). It was then finished and cemented with eugenol-free temporary cement (Provicol; VOCO GmbH, Cuxhaven, Germany). The final indirect restorations were digitally designed, milled, and polished according to the manufacturer’s instructions. The fitting surfaces were sandblasted with 50-µm aluminum oxide particles at a pressure of 2 bar for 15 s as suggested by the manufacturer, and subsequently cleaned in an ultrasonic cleaner (BioSonic, Coltene/Whaledent, USA).

#### Cementation of indirect restorations

Under rubber dam isolation, the provisional restoration was removed, and any temporary cement residues were carefully cleaned using a scaler (Woodpecker, Mident Industrial Co., Ltd., Henan, China). Adjacent teeth were protected with sterilized Teflon tape. The sandblasted intaglio surface of the onlay was silanated with Ceramic Bond (VOCO GmbH, Cuxhaven, Germany). Tooth surfaces were prepared by air-borne particle abrasion with silica-coated aluminum oxide (CoJet Sand; 3 M ESPE, Deutschland GmbH, Germany). This was performed for 4 s at 2 bar pressure with a nozzle angle of 45 ^o^ from a distance of approximately 10 mm using a JEEP Dental Air Prophy/Air device [8, 34, 35, 37]. Finally, the enamel margins of the prepared cavity were selectively acid-etched with 37% phosphoric acid (Ivoclar Vivadent, Amherst, NY, USA) for 30 s [38, 39].

**Fig. 2:**
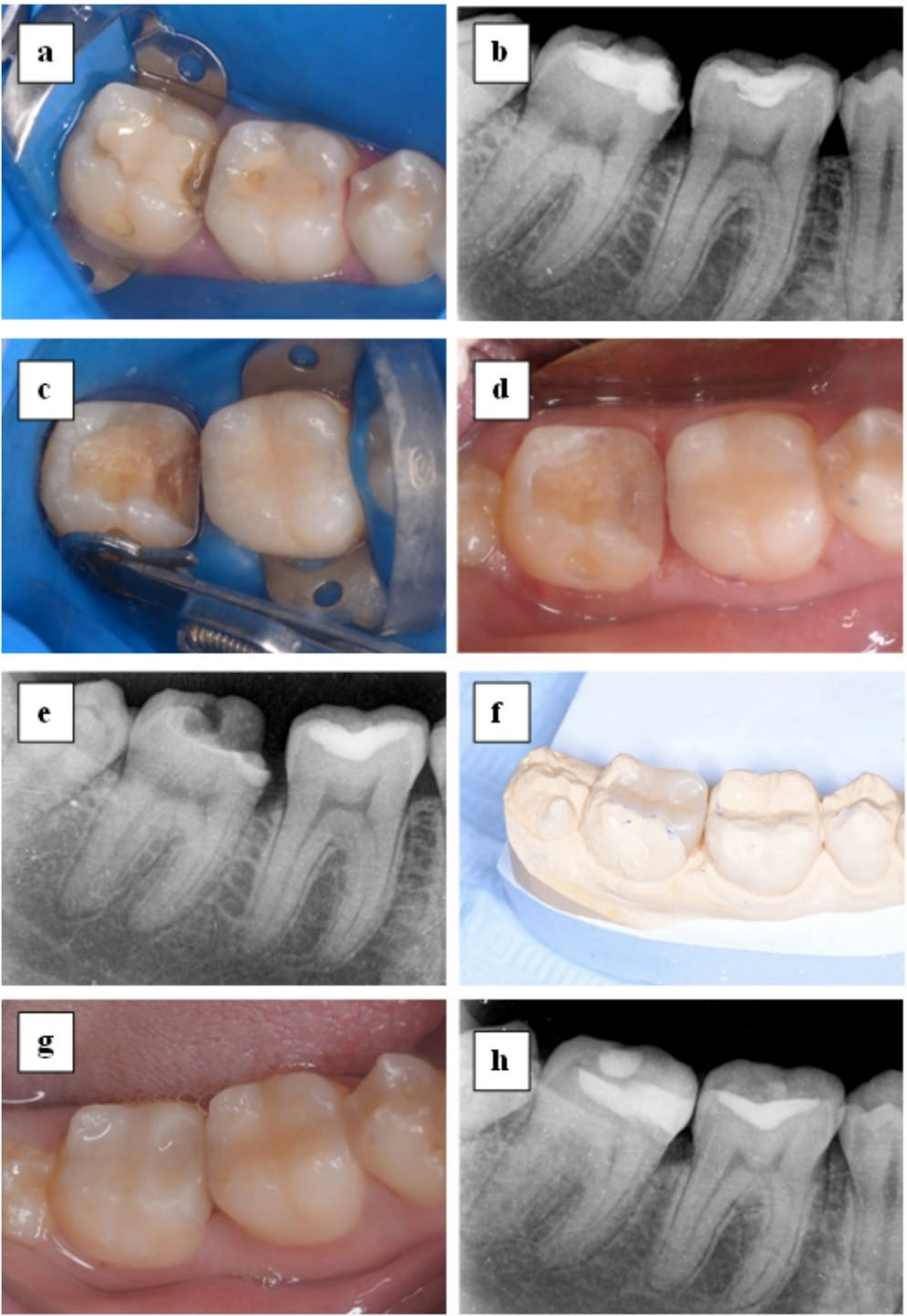
Composed figure representing the clinical procedures for tooth no. 47 **a**: preoperative photograph. **b**: preoperative radiograph. **c**: matrix application for deep margin elevation. **d**: Photograph after deep margin elevation. **e**: Radiograph after deep margin elevation. **f**: Nano-ceramic resin CAD/CAM onlay restoration. **g**: Photograph after onlay cementation. **h**: Radiograph after onlay cementation

The onlay restoration was cemented to the tooth under steady light pressure using self-adhesive, dual-cured resin cement (Bifix SE, VOCO GmbH, Cuxhaven, Germany) according to the manufacturer’s instructions. Fine adjustments were made using fine-grit diamond instruments (Diatech, Coltene, Switzerland), and polishing was performed with a Dimanto Diamond Polisher (VOCO GmbH, Cuxhaven, Germany), as recommended by the manufacturer. Clinical procedures are demonstrated in representative photo- and radio-graphs (Fig. 2).

### Clinical evaluation

Two independent examiners conducted the clinical evaluations, remaining blinded to the type of restoration and study group. Examiner training and calibration were performed by clinically assessing 40 restorations in volunteers not included in the study, using the selected evaluation criteria. An intra-examiner and inter-examiner agreement of at least 84% was required for calibration to be considered satisfactory.

Gingival health was assessed using the GI described by Löe and Silness, which served as the primary outcome [26, 40]. Normal gingiva without inflammation, discoloration, or bleeding was scored 0. Mild inflammation with a slight color change and edema, but no bleeding on probing, was scored (1) Moderate inflammation, characterized by redness, edema, and glazing with bleeding on probing, was scored (2) Severe inflammation, defined as marked redness, edema, or ulceration with a tendency for spontaneous bleeding, was scored 3.

Additionally, restorations were evaluated for clinical performance. The FDI 2010 criteria [41] were modified by using selected functional and biological parameters, and served as the secondary outcome (Table 2). Clinical assessments were recorded at one-week post-onlay cementation (baseline) and at 1-, 2-, and 3-year follow-up periods.

**Table 2:** Modified FDI 2010 Scoring criteria

Evaluation Criteria		Score				
		**1. Clinically very good**	**2. Clinically good**	**3. Clinically satisfactory**	**4. Clinically unsatisfactory**	**5. Clinically poor**
Functional Properties	**Fracture of material and retention**	-No fractures or cracks.	-Hairline crack.	-More than one hairline crack. -larger crack. -material shipping other than margin or contact area.	-material shipping including margin or contact area. -partial loss of less than half of restoration.	-multiple fractures. -loss of more than half of the restoration -complete loss of the restoration.
	**Marginal adaptation**	-No gaps or white or discolored marginal line	-White line or Marginal-gap less than 150-µm -Small marginal ditching, steps or irregularities	-Marginal-gap less than 250-µm - major marginal ditching, irregularities or steps	-Marginal-gap more than 250-µm - severe marginal ditching, irregularities or steps	-Major Marginal-gaps (generalized) -Loose restoration
	**Radiographic examination**	-Smooth transition between tooth and restoration -No pathologic changes	-acceptable excess material -positive or negative step transition between tooth and restoration (less than 150-µm)	-poor radio-opacity of restoration - positive or negative step transition between tooth and restoration (less than 250-µm) -Marginal-gap less than 250-µm	-Marginal-gap more than 250-µm - positive or negative step transition between tooth and restoration (more than 250-µm) -Accessible material excess.	-Secondary caries or apical pathologic changes. -Restoration or tooth fracture or loss.
Biological Properties	**Postoperative hypersensitivity and tooth vitality**	-No hypersensitivity -normal vitality	-minor hypersensitivity for limited time period -normal vitality	-moderate hypersensitivity -delayed sensitivity without complaints	-severe hypersensitivity -delayed sensitivity with minor complaints.	-severe pulpitis or non-vital tooth
	**Recurrence of caries**	-no caries	-localized demineralization	-large demineralization areas	-localized cavitated caries accessible for repair	-deep caries or exposed dentin not accessible for repair
	**Tooth integrity**	-tooth integrity	-small enamel margin fracture or hairline crack (less than 150-µm)	-enamel margin defect or crack less than 250-µm	-Large defect of enamel margin or crack more than 250-µm	-fracture of cusp or tooth

### Statistical analysis

Data obtained from clinical criteria scoring were tabulated and statistically analyzed using IBM SPSS Statistics software version 26. Data normality was assessed using the Shapiro-Wilk test, which indicated a non-parametric distribution. The level of significance was set at *p* ≤ 0.05.

## Results

### Primary outcome (GI)

The Friedman test revealed significant differences between the follow-up periods within each group. Multiple paired Wilcoxon Signed-Rank tests showed that baseline scores were significantly lower than the scores at 1 year, 2 years, and 3 years in all groups. The Kruskal–Wallis test indicated no significant differences between groups at each evaluation period. All data are presented in Table 3.

**Table 3:** Results of gingival index evaluation

Material/Group	Follow up period	GI Score			Friedman test (P*)	Sig.**
			1	2		
HVGI Group	Baseline	13	3	0	P* = 0.001	a
	1-year	8	6	2		b
	2-year	8	5	3		b
	3-year	8	4	4		b
ICR Group	Baseline	15	1	0	P* = 0.001	a
	1-year	10	5	1		b
	2-year	9	6	1		b
	3-year	9	4	3		b
RMGI Group	Baseline	13	3	0	P* = 0.001	a
	1-year	9	5	2		b
	2-year	8	6	2		b
	3-year	6	6	4		c
BAIR Group	Baseline	14	2	0	P* = 0.001	a
	1-year	9	6	1		b
	2-year	7	7	2		b, c
	3-year	7	6	3		c
Kruskal–Wallis test (P***)	*** *P*1 = 0.706*P*2 = 0.892 *P*3 = 0.884 *P*4 = 0.826					

* Friedman testing the results of different follow up periods in the same material group

** Multiple testing different follow up periods results within material group by Wilcoxon Signed Ranks Test

*** Kruskal–Wallis testing the results of different group materials within same follow up period; *P*1: within baseline period, *P*2: within 1-year period, *P*3: within 2-year period, *P*4: within 3-year period.

*p*< 0.05 denotes significant difference

### Secondary outcome (Restorative Assessment)

The frequency and percentages of different recorded scores for groups across the modified FDI criteria are displayed in Table 4. None of the cases received a score of 4 or 5. All evaluated FDI criteria showed no statistically significant differences between the different groups within the same evaluation period.

**Table 4:** Results of selected FDI evaluation criteria

Evaluation			Baseline				1-year				2-year				3-year			
Criteria		score	HVGI	ICR	RMGI	BAIR	HVGI	ICR	RMGI	BAIR	HVGI	ICR	RMGI	BAIR	HVGI	ICR	RMGI	BAIR
			No. (%)	No. (%)	No. (%)	No. (%)	No. (%)	No. (%)	No. (%)	No. (%)	No. (%)	No. (%)	No. (%)	No. (%)	No. (%)	No. (%)	No. (%)	No. (%)
Functional	**Fracture of material and retention**	1	16 (100)	16 (100)	16 (100)	16 (100)	16 (100)	16 (100)	16 (100)	16 (100)	16 (100)	16 (100)	16 (100)	16 (100)	16 (100)	16 (100)	16 (100)	16 (100)
		***p value***	* P = 1.0				* P = 1.0				* P = 1.0				* P = 1.0			
			** P1 = 1.0, P2 = 1, P3 = 1.0, P4 = 1.0															
	**Marginal adaptation**	1	15 (93.8)	16 (100)	16 (100)	16 (100)	14 (87.5)	16 (100)	15 (93.8)	16(100)	14 (87.5)	15 (93.8)	14 (87.5)	15 (93.8)	13 (81.3)	14(87.5)	13 (81.3)	14 (87.5)
		2	1 (6.25)	0	0	0	2 (12.5)	0	1 (6.25)	0	2 (12.5)	1 (6.25)	2 (12.5)	1 (6.25)	2 (12.5)	2(12.5)	2 (12.5)	1 (6.25)
		3	0	0	0	0	0	0	0	0	0	0	0	0	1 (6.25)	0	1 (6.25)	1 (6.25)
		***p value***	* P = 0.392				* P = 0.285				* P = 0.868				* P = 0.919			
			** P 1 = 0.096, *P* 2 = 0.194, *P* 3 = 0.093, *P* 4 = 0.145															
	**Radiographic examination**	1	14 (87.5)	16 (100)	15 (93.8)	16 (100)	14 (87.5)	16 (100)	15 (93.8)	14 (87.5)	13 (81.3)	16 (100)	14 (87.5)	14 (87.5)	12 (75)	14 (87.5)	13 (81.3)	13 (81.3)
		2	2 (12.5)	0	1 (6.25)	0	2 (12.5)	0	1 (6.2)	2 (12.5)	3 (18.8)	0	2 (12.5)	2 (12.5)	3 (18.8)	2 (12.5)	2 (12.5)	2 (12.5)
		3	0	0	0	0	0	0	0	0	0	0	0	0	1 (6.25)	0	1 (6.25)	1 (6.25)
		***p value***	* P = 0.285				* P = 0.503				* P = 0.392				* P = 0.830			
			** P 1 = 0.066, *P* 2 = 0.194, *P* 3 = 0.145, *P* 4 = 0.072															
Biological	**Postoperative (hyper-sensitivity and tooth vitality)**	1	16 (100)	16 (100)	16(100)	15 (93.8)	16 (100)	15 (93.8)	16 (100)	16 (100)	16 (100)	16 (100)	16 (100)	16 (100)	16 (100)	16 (100)	16 (100)	16 (100)
		2	0	0	0	1(6.2)	0	1(6.2)	0	0	0	0	0	0	0	0	0	0
		***p value***	* P = 0.392				* P = 0.392				* P = 1.0				* P = 1.0			
			** P 1 = 1.0, *P* 2 = 0.392, *P* 3 = 1.0, *P* 4 = 0.392															
	**Recurrence of caries**	1	16 (100)	16 (100)	16 (100)	16 (100)	16 (100)	16 (100)	16 (100)	16 (100)	16 (100)	16 (100)	16 (100)	16 (100)	16 (100)	16 (100)	16 (100)	16 (100)
		***p value***	* P = 1.0				* P = 1.0				* P = 1.0				* P = 1.0			
			** P 1 = 1.0, *P* 2 = 1, *P* 3 = 1.0, *P* 4 = 1.0															
	**Tooth integrity (enamel cracks**,** tooth fractures)**	1	16 (100)	16 (100)	16 (100)	16 (100)	16 (100)	16 (100)	16 (100)	16 (100)	16 (100)	16 (100)	16 (100)	16 (100)	16 (100)	16 (100)	16 (100)	16 (100)
		***p value***	* P = 1.0				* P = 1.0				* P = 1.0				* P = 1.0			
			** P 1 = 1.0, *P* 2 = 1, *P* 3 = 1.0, *P* 4 = 1.0															

* Testing results of different material groups within same follow up period by **Kruskal–Wallis’ test**

** Testing results of different follow up periods in the same material group by **Friedman test;** ***P*1**: difference between follow up periods within HVGI group, ***P*2**: difference between follow up periods within ICR group, ***P*3**: difference between follow up periods within RMGI group, ***P*4** difference between follow up periods within BAIR group

**p** < 0.05 denotes significant difference

## Discussion

The optimal restorative approach for managing extensive subgingival Class II carious lesions remains a matter of controversy. Consistent with the principle of minimal invasiveness, there has been a growing shift toward using DME materials beneath partial indirect esthetic restorations [1, 5, 21]. Given that injectable restorative materials offer a simplified solution for these challenging conditions, this study evaluated their impact as DME materials on the clinical performance of nanoceramic resin CAD/CAM onlay restorations.

Randomized controlled trials are considered the gold standard for establishing causality in evidence-based research. Designing such trials requires careful planning and coordination, acknowledging that no scientific methodology is without limitations [20]. To minimize the risk of misleading results, this randomized clinical trial was planned and conducted in accordance with the CONSORT guidelines [29]. A multi-arm parallel design was adopted due to the difficulty of recruiting patients who met al.l inclusion criteria across the four oral quadrants.

Regarding DME, the distance between the cervical margin and the alveolar bone crest is critical for preserving supracrestal tissue attachment and periodontal health. The biological width is commonly reported as approximately 2.04 mm, although significant variations have been documented [42]. Traditionally, crown lengthening was recommended when the distance between the alveolar bone crest and the restoration margin was less than 3 mm to avoid violating the biological width [43]. Studies have shown that a well-adapted subgingival composite margin, which does not invade more than 1.07 mm of connective tissue attachment, is generally well tolerated by periodontal tissues [44–46]. Following the minimally invasive approach, this study excluded cases in which the distance between the sound proximal cervical margin and the alveolar crest was less than 2 mm to minimize the variability in periodontal state [26, 47, 48].

Dual-cured resin cements are recommended for cementing thick partial indirect restorations, such as inlays or onlays. Although a chemical reaction initiates the setting of the cement, light activation is required to achieve maximum polymerization [49, 50]. In this study, a dual-cured self-adhesive resin cement was used to ensure complete polymerization even at the cavity base, while the self-adhesive property standardized the cementation procedure. Selective enamel etching was performed additionally to enhance bonding, addressing the weaker adhesion of self-adhesive cements to enamel [38, 39].

Gingival health was considered the primary clinical evaluation parameter in this study, as restorative materials in contact with gingival tissues can compromise their health [51]. The response of gingival tissues to subgingival restorations is influenced by multiple factors, including restoration contour and margins, as well as iatrogenic issues such as overhangs, marginal discrepancies, and the type of restorative material used [52]. The Gingival Index was used to assess periodontal health, as gingival inflammation is a key indicator of periodontal disease [53].

A clinical restorative evaluation was conducted over three years, representing a medium-term assessment. Since all study groups received the same nanoceramic resin CAD/CAM onlay material, the selected FDI criteria excluded parameters evaluating only the overlying restorations, such as esthetic appearance, occlusal wear, and proximal contour. Instead, criteria addressing restorative success, adaptation, and performance of the different cervical lining materials were included. Radiographic evaluation was performed despite standard radiation protection recommendations [54], as it provided valuable information on subgingival tooth-restoration margins that were difficult to assess clinically after gingival healing had occurred. Radiographic scoring was based on the detection of pathological or restorative defects and the adaptation of the DME material to the cervical margin.

The baseline GI scores were lower than the follow-up scores for all material groups, and no cases exhibited severe gingival inflammation. These results align with previous studies [26, 55] that report increased gingival bleeding on probing with subgingival restoration margins. Furthermore, Muscholl [55] emphasized the benefit of daily interdental brush use in reducing gingival inflammation. In contrast, Bertoldi [23] reported decreased gingival bleeding adjacent to DME restorations, which was comparable to that of control non-restored teeth at the three-month evaluation. These differing outcomes may be attributed to variations in study design and inclusion criteria. In Bertoldi’s study, DME was limited to restorative margins at least 3 mm from the alveolar crest, following the traditional concept. In the present study, the more conservative approach with margins 2 mm from the alveolar crest [26, 48], combined with individually impaired oral hygiene, may have contributed to the observed increase in gingival bleeding [56].

Violation of the biological width can lead to gingival and periodontal inflammation, manifesting as gingival redness, bleeding, pain, pocket formation, and loss of clinical attachment and bone [23]. However, subgingival DME restorations with smooth, well-finished surfaces can create a favorable environment for bone and soft tissues, allowing a longer junctional epithelium along the restoration and a smaller connective tissue attachment [1, 57]. These conditions are generally well tolerated by soft tissues, and good oral hygiene further supports periodontal health.

Evaluation of GI scores between different materials within each period yielded comparable results with no notable variation. No studies have directly compared various DME materials beneath vital posterior partial indirect restorations. The findings of this study are consistent with those of Ismail et al. [27, 58], who assessed periodontal health using different DME materials (glass hybrid, resin-modified glass ionomer, bulk-fill composite, and bioactive ionic resin) beneath direct composite restorations, reporting comparable scores across materials in 2- and 3-year follow-ups.

Evaluation of the modified FDI criteria revealed comparable scores between material groups within each period and across different periods within each group. All assessed functional and biological criteria were recorded as successful, resulting in a 100% success rate, with no cases receiving grades of 4 or 5. These results align with previous in vivo studies [27, 55, 59] reporting clinically successful FDI scores for DME restorations. Eventually, since the clinically evaluated outcomes demonstrated successful results across all groups, the null hypothesis was accepted.

Despite careful planning, this clinical trial had several limitations. Evaluating the periodontal probing depth would provide precious insight into the periodontal health. Moreover, bitewing radiographs could provide more precise standardized data for determining the distance between the cervical margin and the crestal bone. Correlating bone levels with periodontal health throughout the follow-up period would be valuable to assess the clinical attachment level. Additionally, a more extended evaluation period is required to detect potential differences between DME materials. Furthermore, despite encouraging participants to maintain optimal oral hygiene throughout the study, compliance with these measures remains a great concern.

## Conclusions

Within the limitations of this study, it was concluded that the use of injectable restorative materials for deep margin elevation did not adversely affect the three-year clinical performance of CAD/CAM resin-based onlay restorations. However, this procedure may result in a slight increase in gingival bleeding.

## Supplementary Information

Below is the link to the electronic supplementary material.

Supplementary Material 1 (DOC 232 KB)

## Data Availability

The datasets used and/or analyzed during the current study are available from the corresponding author on reasonable request.
